# Specific expression of APOE in astrocytes reduces Aβ accumulation and plaque‐related pathology in a mouse model of amyloidosis

**DOI:** 10.1002/alz.090129

**Published:** 2025-01-03

**Authors:** NA WANG, Sydney V. Doss, Yuzhou Chang, William Tauer, Yingxue Ren, Fangfang Qi, Guojun Bu, Chia‐Chen Liu, Long‐Jun Wu

**Affiliations:** ^1^ Mayo Clinic, Rochester, MN USA; ^2^ Mayo Clinic, Jacksonville, FL USA

## Abstract

**Background:**

Accumulation of the amyloid‐β (Aβ) peptide into amyloid plaque is one of the key pathological markers of Alzheimer’s disease (AD). Apolipoprotein E (APOE) is known to modify AD risk and has been reported to influence Aβ accumulation in the brain in an isoform‐dependent manner. ApoE can be produced by various cell types in the brain, with astrocytes being the main producer. Increasing studies show that altering apoE levels can influence Aβ plaque pathology. However, it is not fully understood how apoE produced by specific cell types, such as astrocytes, contributes to amyloid pathology.

**Method:**

We utilized mouse models expressing APOE isoforms in astrocytes and crossed them with 5xFAD mouse models of amyloidosis. We analyzed the changes to amyloid plaques and assessed the impact on cellular responses to amyloid plaques in condition of astrocytic APOE expression.

**Result:**

Specific expression of APOE3 in astrocytes resulted in compact amyloid plaques and a large increase in plaque deposition, while the total plaque burden was reduced. Astrocytic APOE2 expression led to similar changes to amyloid plaques, but the fold change is lower comparing to that in astrocytic APOE3 models. Intriguingly, the total microglial response increased dramatically in condition of astrocytic APOE3 expression but not in condition of astrocytic APOE2 expression. Further, the ratio of plaque associated Lgals3/Iba1 is higher in condition of astrocytic APOE3 expression, indicating increased microglial activation. Additionally, astrocyte GFAP levels only increased in astrocytic APOE3 models, but not in astrocytic APOE2 models. Finally, APOE isoform effect on dystrophic neurites amount was also observed.

**Conclusion:**

Together, our study reveals an important role of astrocytic APOE on the deposition and accumulation of Aβ plaques as well as on Aβ‐associated downstream effects.

